# Electric Bicyclist Injury Severity during Peak Traffic Periods: A Random-Parameters Approach with Heterogeneity in Means and Variances

**DOI:** 10.3390/ijerph182111131

**Published:** 2021-10-22

**Authors:** Tong Zhu, Zishuo Zhu, Jie Zhang, Chenxuan Yang

**Affiliations:** 1 College of Transportation Engineering, Chang’an University, Xi’an 710064, China; zhutong@chd.edu.cn; 2 Research Institute of Highway, Ministry of Transport, Beijing 100088, China; zhangjiebit@126.com; 3 Department of Civil, Construction and Environmental Engineering, The University of Alabama, Tuscaloosa, AL 35487, USA; cyang30@crimson.ua.edu

**Keywords:** mixed logit model, heterogeneity in means and variances, injury severity, electric bicycle crashes, visibility

## Abstract

Accidents involving electric bicycles, a popular means of transportation in China during peak traffic periods, have increased. However, studies have seldom attempted to detect the unique crash consequences during this period. This study aims to explore the factors influencing injury severity in electric bicyclists during peak traffic periods and provide recommendations to help devise specific management strategies. The random-parameters logit or mixed logit model is used to identify the relationship between different factors and injury severity. The injury severity is divided into four categories. The analysis uses automobile and electric bicycle crash data of Xi’an, China, between 2014 and 2019. During the peak traffic periods, the impact of low visibility significantly varies with factors such as areas with traffic control or without streetlights. Furthermore, compared with traveling in a straight line, three different turnings before the crash reduce the likelihood of severe injuries. Roadside protection trees are the most crucial measure guaranteeing riders’ safety during peak traffic periods. This study reveals the direction, magnitude, and randomness of factors that contribute to electric bicycle crashes. The results can help safety authorities devise targeted transportation safety management and planning strategies for peak traffic periods.

## 1. Introduction

Peak periods have the highest probability of road accidents worldwide. A high traffic flow, riders’ eagerness to reach their destination, and the pressure of congestion contribute to the likelihood of accidents during this period. Consequently, the number of crashes occurring during peak hours is dramatically higher than in off-peak hours [1]. Existing studies on peak periods tend to focus on automobile driver injury severity on highways [2] and in rural areas [3,4,5]. Some studies also highlight other unique indicators related to peak periods, such as driver distraction [6] and traveler choice [7]. Accordingly, the traffic management must be trained and the relevant facilities upgraded with respect to the characteristics and influencing factors of this period rather than those of the off-peak period. To ensure the safety of commuters, the actual factors affecting the crash consequences and passengers’/riders’ injury severity during the peak traffic periods must be considered. However, most studies are focused on evaluating traffic safety at different hours of a day, rather than in peak hours. Consequently, it has been difficult to recommend optimal safety guidelines and facilities for peak traffic periods.

Electric bicyclists represent a sizable population of commuters [8,9,10]. By 2019, there were 59 electric bicycles for every 100 households in China [11]. Bicyclists accounted for 26% of all deaths worldwide [12]. According to the China Traffic Management Bureau, China had 250 million electric bikes (e-bikes) in 2017. Meanwhile, from 2013 to 2017, e-bike-related crashes resulted in approximately 56,200 injuries and 8431 fatalities in the country. The traffic safety of e-bikes cannot be ignored [13]. Among the fatal crashes involving electric bicycles, automobiles accounted for 71.01% [14], which indicates that crashes involving electric bicycles and automobiles deserve more study.

This study aims to determine the factors affecting injury severity among electric bicyclists during peak traffic hours. Based on the factors covered in previous works, this study considered the characteristics of automobile drivers, electric bicyclists, roads, and circumstances, as well as vehicle performance. It also considered vehicle maneuvers (traveling straight, U-turns, and turning left and right) before crashes because these reflect the motivation of riders to illustrate the cause of the crash from the perspective of automobile drivers [15,16]. This study used mixed logit models with heterogeneity of means and variances. The data for analysis were extracted from crash incidents involving electric bicycles from 2014 to 2019 in typical large- and medium-sized cities in China. To the best of the authors’ knowledge, this study is the first to analyze injury severity among electric bicyclists during peak traffic periods.

## 2. Literature Review

### 2.1. Traffic Safety during Peak Traffic Periods

In Table 1, ordered by year of publishing, studies that considered the peak traffic period focused mainly on the crash risk or consequences produced per hour or over other periods [4,5,6,7,17,18,19,20]. In a single-vehicle model, the injury severity among drivers meeting with accidents on rural highways during the busy harvest periods was found likely to be non-incapacitating [21]. However, no studies have examined the performance and injury severity among electric bicyclists meeting with accidents during peak traffic periods.

### 2.2. Traffic Safety of Electric Bicycles

Table 2 summarizes the literature on electric bicyclists’ injury investigations. The table uses labels to identify studies that considered or discovered heterogeneity of parameters (affected by unobserved factors; the same indicator may produce different impacts on the dependent variable). Previous studies have shown that electric bicyclists were more prone to fatal injuries than traditional bicyclists [22,23,24,25,26]. In addition to using IMPACT, a finite element analysis tool, to recreate accident scenes [9,22,27], researchers used statistical models to analyze the factors affecting injury severity among electric bicyclists [10,14,24,25,26]. Characteristics pertaining to humans, vehicles, roads, and circumstances influence the injury severity. As observed in Table 2, studies have demonstrated the different effects of unique factors in addition to sociodemographic characteristics. However, few have laid emphasis on collecting data from a moving vehicle before the crash, and the specific road and visibility conditions at the time of the incident. These factors cannot be dismissed during peak traffic periods, and their relationships with peak-hour crashes warrant further investigation.

### 2.3. Heterogeneity of Crash Models

Despite the various performance metrics of a rider causing varying degrees of injury, most data analyses on injury severity utilized conventional models to simulate severity. Thus, the severity is often underestimated or overestimated [28]. For example, in a study on motorcycle injury severity, researchers used a multinomial logit model to examine the severity, as it was divided into different levels [29]. However, this model was prone to violating the independence of the irrelevant alternative property. Subsequently, nested and ordered logit models appeared to have solved this problem [30]. However, owing to limited data availability, analysts could not obtain all the factors related to a victim’s injury severity. Therefore, it is important to use an effective approach that can capture implicit characteristics so that analysts can understand the crucial relationships among the known indicators and their effects on the subjects (injury severity).

Accordingly, a few studies have constructed models to elucidate the obscure heterogeneity of parameters in the analysis of electric bicycle crash data [31,32,33,34,35]. The mixed logit model with heterogeneity in means (and variances) explains heterogeneity at the individual level [15,16,34]. By relaxing the limitation of fixed parameters, this model performs better than the traditional logit model and requires fewer crash data [36]. The Markov switching model establishes heterogeneity due to the time span [37,38]. Latent-class models are used to illustrate the heterogeneity at the group level [32,39,40]. Furthermore, classical models with random parameters can explain the phenomenon of heterogeneity to a certain extent, such as bivariate or multivariate models with random parameters [41,42], generalized ordered probability models with random parameters of heterogeneity in means and variances [43], ordered probit models with random parameters of heterogeneity in means and variances [33], and random thresholds random parameters hierarchical ordered probit models [44,45].

These studies indicate that peak traffic periods have a significant impact on injury severity. Furthermore, electric bicyclists are facing increasingly challenging traffic scenarios and traffic conflicts. To improve traffic safety, Fyhri evaluated traffic safety of electric bicyclists [24]. However, the study did not focus on the peak traffic periods; it aimed to evaluate the behavioral patterns of electric bicyclists through three models: a mixed logit model, a model with heterogeneity in means, and one with heterogeneity in means and variances. Thus, it overcame the limitation of implicit heterogeneity in the crash data by capturing the heterogeneity in the means and variances of random parameters [28,46].

## 3. Data Description

We collected automobile and electric bicycle crash data of a typical city in China from 2014 to 2019. According to Downs (2005), peak-hour or rush-hour congestion occurs between 6 a.m. and 9 a.m. and again between 4 p.m. and 7 p.m. Based on congestion data crawling and a common work routine, we found that a typical city’s commuter congestion (red area in real-time traffic flow conditions) was in accordance with Downs’s study [17]. Thus, we divided the crash time of day into different segments and then extracted the crashes that occurred between 6 a.m. and 9 a.m. and 4 p.m. and 7 p.m. For data integrity and availability, the study extracted the single-automobile—single-electric bicycle crashes that occurred in the morning and evening hours (2025). Of those crashes, only 998 resulted in property damage as the most severe outcome (hereinafter referred to as no-injury), 596 crashes resulted in minor injury, 324 in severe injury, and 107 in fatal crashes. Each observation of the dataset contained the electric bicycle injury severity and driver and rider characteristics, vehicle characteristics, and road characteristics that influenced the crashes. Table 3 lists the results of the descriptive statistical analysis of the peak traffic model. However, this database does not include all the factors that may contribute to electric bicycle crashes. As an important indicator, *visibility* is the maximum distance up to which a rider can see under natural obstacles like haze and heavy fog rather than the visual distance affected by surrounding vehicles or buildings. This indicator was considered because crashes during haze or fog are common in many provinces in China, including Xi’an. Its influence on accidents during peak hours shall be further discussed. In Xi’an, the local traffic police officers use measurement instruments under haze and heavy fog, or visually record visibility under pleasant weather conditions with no haze or heavy fog, and upload the data to a centralized database. It is noted that specific speed data was important in the previous studies but excluded in this study, because the data was estimated manually by the local policemen. The subjective estimation is unreliable which may bias the model results. As a result, the speed variable was excluded in this study. However, to address the speed indicator, the study considered the rider’s maneuver of braking before crash. These data were collected from the riders, and policemen, who judged the pedals’ final status.

**Figure 1:**
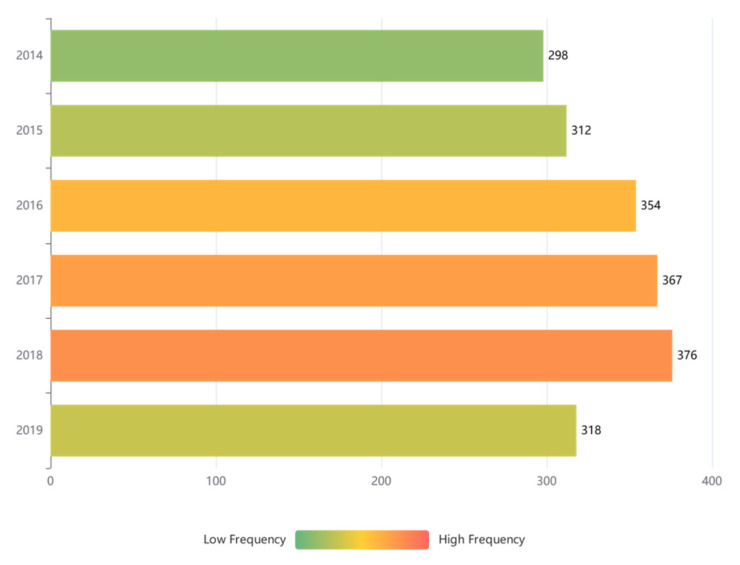
Crash frequency vs. time.

Figure 1 presents the trend of crashes over ten years. A significant rise in peak traffic crashes can be observed between 2014 and 2018. It is a record of all crashes that incurred a property loss of more than CNY 5000 or minor, severe, or fatal injuries to the electric bicyclist. The *x*-axis represents the crash frequency each year, while the *y*-axis represents time. The number of crashes in 2018 surged by approximately 26% in comparison to 2014, probably due to the drastic increase in the demand for electric bicycles. The crash frequency showed a minor increasing trend from 2014 to 2018, and then the frequency declined moderately.

## 4. Methodology

To investigate the implicit heterogeneity in electric bicycle and automobile crash data, the study adopted the method of Seraneeprakarn [28], which is based on an investigation of the random parameters approach with heterogeneity in means and variances. The method relaxes the restriction on assuming the random parameter means and variances for all observations to track the different effects of the studied indicators on varying observations.

First, the injury severity was divided into four categories: no injury (only property loss), minor injury (visible but non-incapacitating injury), severe injury (incapacitating injury), and fatal injury (injury leading to death). No injury was selected as the baseline because it occupied the largest proportion among the four severity scales. This ensured the model produced stable estimations [47]. We defined an injury severity determination function as follows:

(1) $$F_{ik}=\beta_{ik}X_{ik}+\varepsilon_{ik}$$

where $F_{ik}$ represents the injury severity level i (i = 1—no injury: baseline; 2—minor injury; 3—severe injury; and 4—fatal injury) of an electric bicyclist k, $X_{ik}$ is the studied indicator related to the severity, and $\beta_{i}$ is the effect estimator. The error term $\varepsilon_{ik}$ captures the implicit effects or characteristics assumed to have a generalized extreme-value distribution.

Based on the study of Behnood and Mannering [16], $\beta_{i}$ is the key parameter to capture the heterogeneity in the mean and variance of random parameters, which is expressed as follows:

(2) $$\beta_{ik}=\beta +\theta_{ik}Z_{ik}+\sigma_{ik}EXP{\left(\omega_{ik}W_{ik}\right)}_{\gamma_{ik}}$$

where *β* is the mean parameter estimator across all crashes, and $Z_{ik}$ and $W_{ik}$ are vectors that track the heterogeneity in mean and standard deviation (SD) $\sigma_{ik}$. *ω_ik_* is the corresponding parameter vector. $\theta_{ik}$ is a vector corresponding to the estimated parameter $X_{ik}$, and *γ_ik_* is the disturbance term.

To estimate the probability of an electric bicyclist suffering an injury of one of the severity levels, with *ε_ik_* having a generalized extreme value distribution, the choice probability was extended to the multinomial logit model with heterogeneity observation (mixed logit model) formula [47]:

(3) $$P_{n}\left(i|\phi \right)=\int^{\;}\frac{EXP\left[\beta_{ik}X_{ik}\right]}{\sum_{i\epsilon I}EXP\left[\beta_{ik}X_{ik}\right]}f\left(\beta_{ik}|\phi \right)d\beta_{ik}$$

where $P_{n}\left(i|\phi \right)$ is the probability of an electric bicyclist suffering an injury severity level *i* on $f\left(\beta_{ik}|\phi \right)$, $f\left(\beta_{ik}|\phi \right)$ is the density function of $\beta_{ik}$ required to determine $\beta_{ik}$, which can be used to observe the heterogeneity [2]. *ϕ* is the vector of a usual and known density function. In the study, the maximum likelihood estimation with Halton draws was used for the mixed logit model [48].

Two tests were conducted to validate the peak traffic period model. The first one was the log-likelihood test between the overall model and the peak traffic period model [49], which is as given as follows:

(4) $$LR_{overall}=-2\left[LL\left(\beta^{overall}\right)-LL\left(\beta^{traffic\;peak}\right)-LL\left(\beta^{off-peak}\right)\right]$$

where $LR_{overall}$, $LL\left(\beta^{traffic\;peak}\right)$, and $LL\left(\beta^{off-peak}\right)$ are the log-likelihoods at the convergences of the models estimated with data from both peak traffic and off-peak periods, peak traffic period, and non-peak traffic period, respectively. In the first test, the three models (overall model, peak traffic period model, and off-peak model) had the same variables. $LR_{overall}$ is the chi-square ($\chi^{2}$) distributed with degrees of freedom equal to the summation of the number of estimated parameters in the peak traffic and off-peak models minus the number of estimated parameters in the overall model.

Based on the results of the first test, a second test, called the parameter transferability test [49], was conducted to determine whether the peak traffic period was to be modeled separately:

(5) $$LR_{a_{b}}=-2\left[LL\left(\beta_{a_{b}}\right)-LL\left(\beta_{a}\right)\right]$$

where $LL\left(\beta_{a_{b}}\right)$ and $LL\left(\beta_{a}\right)$ are the log-likelihoods at the convergences of the models maintaining converged parameters from the peak traffic period model with the data of the non-peak traffic period and peak traffic period data, respectively. Similarly, $LR_{a_{b}}$ is *χ*^2^ distributed with degrees of freedom equal to the number of estimated parameters in $\beta_{a_{b}}$. The simulation procedure required Halton draws [50].

## 5. Model Estimation Results

To determine if the models need to be developed separately, this study used the econometric analysis software NLOGIT 5.0 (Econometric Software, Inc.: Plainview, NY, USA). The log-likelihood ratio test illustrated a test statistic of 128.77 with 32 degrees of freedom (*p* < 0.001), which implies that the peak traffic period must be modeled separately with a confidence interval of more than 99%. According to the model separation test, each test statistic and the corresponding degrees of freedom suggest that the peak period must be modeled separately among electric bike-involved crashes with more than 99% confidence ($LR_{peak_{off-peak}}$ = 365.2, df = 40;$\;LR_{off-peak_{opeak}}$ = 493.4, df = 43). Halton draws are more effective than random draws in ensuring better convergence with shorter drawing times. In this study, we narrowed the number of Halton draws to 200 for greater fitness and accurate parameter estimation of data. Moreover, in the model estimation, a normal distribution proved to be the best statistical fit for the functional form of the parameter density function, which conforms with previous studies [44,46].

During the model development, the indicators were considered significant if their t-statistics corresponded to the 90% confidence level or higher on a two-tailed t-test. Then, random indicators are addressed by determining their standard deviations to have the t-statistics corresponding to the 90% confidence level or higher [7]. To identify the heterogeneity of the means of random parameters and ensure the t-statistics corresponded to the 90% confidence level or higher, we estimated the parameters and the SDs of random parameters influenced by other non-random parameters. Moreover, identifying the heterogeneity of the means and variances of random parameters involves an additional test of significance of the heteroscedasticity of random parameters under the influences of other non-random parameters. This step requires the heteroscedasticity of random parameters to have t-statistics corresponding to a confidence interval of 90% or higher.

To investigate the heterogeneity in the means and variances of parameters, this study maintained three models based on a mixed logit model with or without consideration of heterogeneity. Table 4 and Table 5 list the estimations of the models and the marginal effects, respectively. No injury is the baseline of the severity outcome. Its severity function is constrained to zero without loss of generality. This study focuses on the injury severity of electric bicyclists rather than automobile drivers because bicyclists are more likely to sustain serious injury.

To identify the best-fit model in this study, Table 6 lists the log-likelihoods at convergence for the constant-only, multinomial logit, mixed logit, and mixed logit with heterogeneity in means and variances models. McFadden ρ^2^ (conditional logarithmic analysis value of qualitative selection behavior) and BIC (Bayesian Information Criterion) determine the goodness of fit of the model. Specifically, the model contains better fitness with higher McFadden ρ^2^ and lower BIC.

The likelihood ratio tests demonstrated the statistical robustness of the mixed logit model with heterogeneity in means and variances, i.e., according to the null hypothesis, this model equals the other models being rejected with more than 99% confidence. The lowest BIC value of the mixed logit model with heterogeneity in means and variances reveals that it is important to capture the sources of heterogeneity in means and variances, thus it was selected as the final model.

The significant parameters and related findings are discussed in the subsequent paragraphs. The parameters that produced arbitrary degrees of injury severity are discussed. To detect the factors associated with the performance of the random parameters, the heterogeneity findings originating from those random parameters are outlined. Observations of other statistically significant parameters grouped by category are also summarized. Finally, the marginal coefficients are separately discussed after estimating their parameters, which directly influence electric bicyclist injury severity. All the variables kept in the models are statistically significant at a 0.10 significance level.

### 5.1. Random Parameters and Heterogeneity Observations

**Figure 2:**
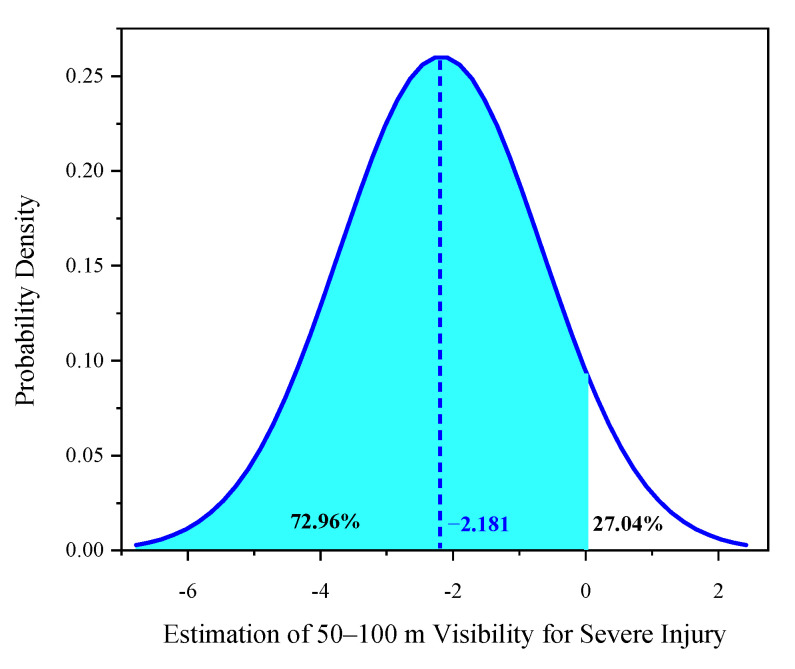
Parameter distribution of 50–100 m visibility indicator for severe injury.

**Figure 3:**
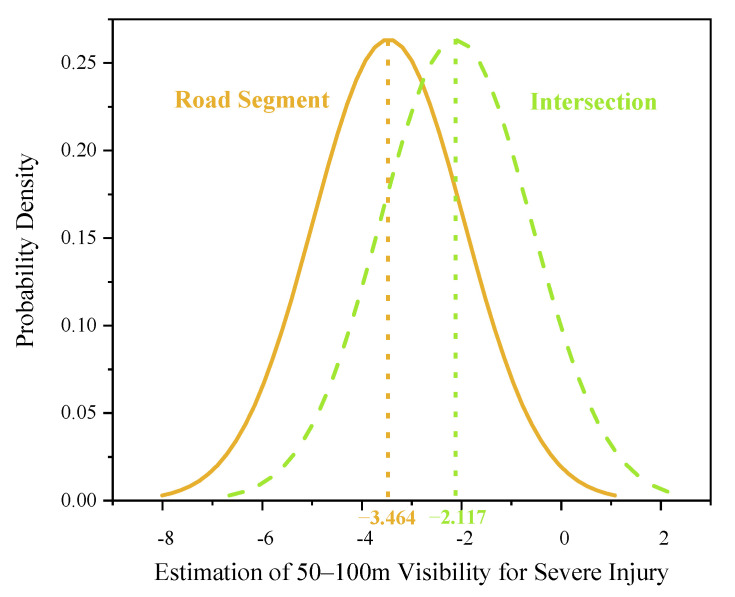
Parameter distribution of 50–100-m visibility indicator for different road types.

**Figure 4:**
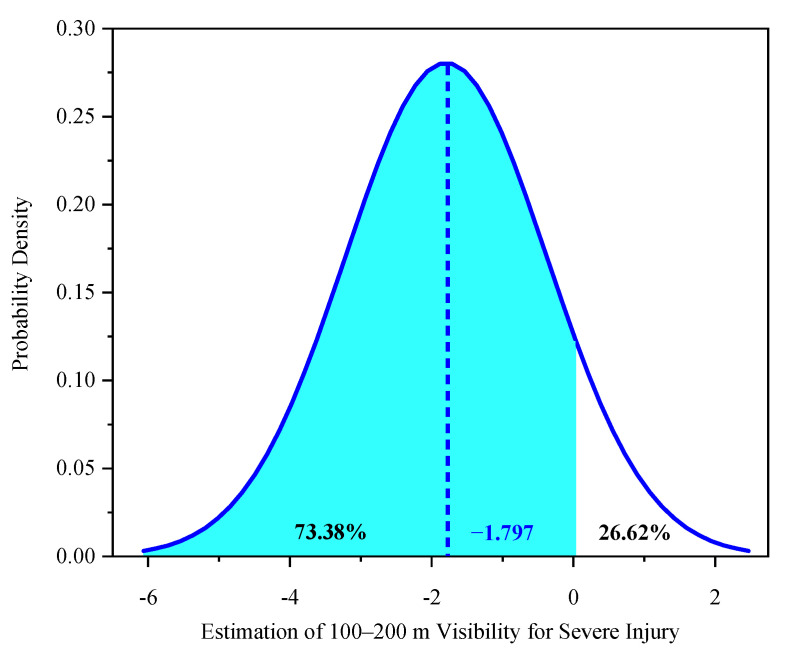
Parameter distribution of the 100–200-m visibility indicator for severe injury.

**Figure 5:**
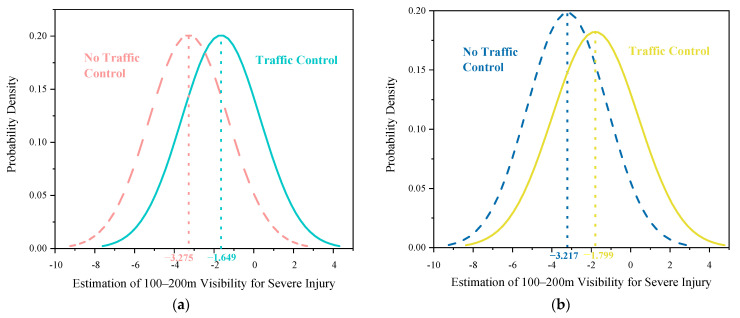
Parameter distribution of 100–200-m visibility indicator under traffic control. (**a**) Mean heterogeneity; (**b**) Mean-variance heterogeneity.

**Figure 6:**
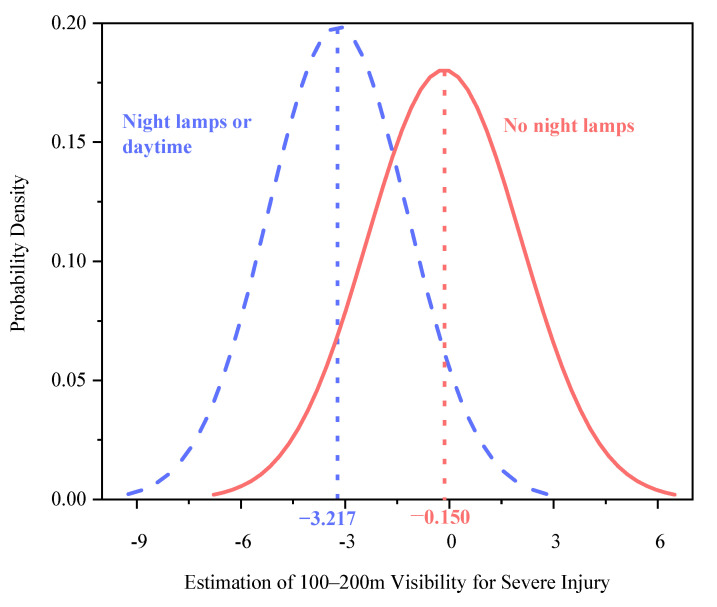
Parameter distribution of 100–200-m visibility indicator without night lighting.

Two explanatory variables were found to be randomly distributed in the mixed logit model: 50–100 m visibility—severe injury and 100–200-m visibility—severe injury (Table 4). The two random parameters only passed the normal distribution test with 95% confidence in the common crash data distribution tests, including normal, uniform, and triangular distributions. The predicted possibilities of each random indicator can be observed visually (Figure 2, Figure 3, Figure 4, Figure 5 and Figure 6). To be specific, the x-axis represents the estimation of the indicator, while the y-axis represents the corresponding possibility.

For the 50–100 m visibility indicator, with a mean of −2.181 and an SD of 2.348, there were approximately 72.96% of crashes with natural visibility of 50–100 m leading to less severe injuries (Figure 2). Poor visibility during peak hours may reduce riders’ line of sight, increasing their cautiousness. Therefore, most riders steadily depressed the brake pedal and decelerated. However, in the other 27.04% of crashes, riders preferred to apply a brake lag or less effective avoidance maneuvers amid haze. Consequently, bicyclists were prone to fall or collide, suffering severe injuries. What is more, heterogeneity in means of this random parameter was observed. Compared with driving at an intersection, driving on road segments with 50–100 m visibility decreased the mean of the 50–100 m visibility range, reducing the probability of severe injury. At intersections (i.e., the reference category) amid visibility of 50–100 m in peak hours, the estimated mean parameter was −2.117. On road segments, the mean parameter was −3.464 (−2.117 − 1.347 = −3.464), as illustrated in Figure 3. Therefore, the probability of severe injury amid 50–100 m visibility decreased during peak hours. Driving on road segments under low visibility motivated electric bicyclists to drive cautiously. Consequently, their cautions will prevent the riders into a serious or severe injury. This is demonstrated by the negative mean further away from zero, which resulted in more riders suffering mild injuries during a crash.

The 100–200 m low-visibility parameter also randomly affected electric bicyclist injuries [7]. Similarly, 73.38% of riders involved in crashes under visibility of 100–200 m did not suffer severe injuries, based on the normal distribution with a mean of −1.797 and an SD of 2.023 (Figure 4). However, the marginal effect of this indicator shows its increasing contribution to less severe injuries among electric bicyclists (Table 5). Similarly, when the visibility was only 100–200 m, riders still preferred to focus and decelerate. However, compared with visibility of 50–100 m, when the range was 200 m, riders were less likely to sustain severe injuries in a collision or fall because they could perform careful maneuvers despite the road conditions. Accounting for heterogeneity in means only, compared with driving in areas without traffic control, driving in areas with traffic control increased the mean of the 100–200 m visibility range, increasing the probability of severe injury. On the one hand, in areas with traffic control, the mean parameter for the 100–200 m visibility range is −1.649 (−3.275 + 1.626 = −1.649). On the other hand, in areas with no traffic control (i.e., the reference category), the estimated mean parameter for the same visibility range—severe injury is −3.275 (Figure 5a).

Accounting for heterogeneity in means and variances, the 100–200 m visibility range—severe injury, was found to produce random parameters with heterogeneity in means and variances. Compared with ‘no traffic control area’ driving, ‘traffic control area’ driving increased the mean of this range to −1.799 (−3.127 + 1.418 = −1.799), increasing the probability of severe injury. However, the mean of visibility in the no-traffic-control scenario was −3.127 (Figure 5b). More importantly, the variance of the 100–200 m visibility indicator increased (4.037^2^ + 0.568 = 16.865 > 4.037^2^).

Road illumination during the evening peak period also influenced the mean and variance of the 100–200 m visibility range. The study demonstrates that the estimated mean parameter for 100–200 m visibility—severe injury, when driving in well-lit areas (daytime, i.e., the reference category) was −3.217. For areas with no nightlights, this parameter became −0.150 (−3.275 + 3.067 = −0.150) (Figure 6). The results suggest that the 100–200 m visibility range decreased the probability of severe injury to electric bicyclists, but the reduction was markedly weakened when driving in areas with no nightlights. This indicates that 100–200 m visibility decreases the possibility of severe injury to the electric bicyclists, albeit at a significantly lower rate in areas with no nightlights. This also means that the lack of sunlight due to haze during the afternoon peak periods reduces drivability and increases the risk of grave accidents. Furthermore, night driving without streetlights increases the variance (4.037^2^ + 0.732 = 17.029 > 4.037^2^) in severe injury (Figure 6). That is, the presence of streetlights during the evening peak hours adversely affects electric bicyclists’ driving performance, which leads to severe injury rates fluctuating more.

The marginal effects show that driving amid visibility of 50–100 m and 100–200 m significantly decreased the likelihood of electric bicyclists suffering severe injury by 0.1824 and 0.1791 (Table 5), respectively. The results indicate that poor visibility is markedly sensitive to heterogeneity in means and variances in terms of grave injury to electric bicyclists (Table 4).

### 5.2. Driver and Bicyclist Characteristics

In terms of human characteristics, ‘female electric bicyclist’ is the only indicator that significantly affected injury severity. The marginal effects showed that female electric bicyclists increased the possibility of injury by 0.0137 (Table 5), which conforms with the findings of previous research on bicyclists’ sex [15,16,31].

### 5.3. Vehicle Characteristics

Among the various types of automobiles, accidents involving passenger cars and trucks contribute to the increasing number of incidents causing severe injuries (Table 4), which conforms with previous studies [4,5,18,21]. However, if a crash occurs between a motorcycle and an electric bicycle, the rider of the latter is more likely to suffer only property damage. The marginal effects indicate an overstatement of the heterogeneity in means and variances in the analysis (0.2711 > 0.1533).

### 5.4. Pre-Crash Vehicle Movement Characteristics

Vehicular movement before a crash influences the injury severity of electric bicyclists. Contrary to popular belief, all maneuvers (U-turn, left turn, and right turn) reduce the possibility of electric bicyclists sustaining severe and fatal injuries compared with traveling in a straight line (Table 4). Specifically, compared with turning left, turning right is the leading cause of death of electric bicyclists (−0.1033, see Table 5). In contrast, a U-turn is less likely to cause the occurrence of fatal injury (−0.0566). Specifically, the most efficient movement to prevent serious injury or fatal is turning right. Conversely, U-turn is proven to be the least efficient movement to prevent serious injury and fatal.

### 5.5. Roadway and Environmental Characteristics

The road conditions and environment also influence the injury severity. In terms of road infrastructure and conditions, despite the presence of traffic signals, border trees, and fences in accident zones, other indicators pose greater risks of serious injury to bicyclists, and include driving on road segments (relative to intersections), graded highways and urban roads, and driving in urban areas.

According to the marginal effects of indicators, the implementation of traffic control measures and the erection of border trees and protective fences reduces the injury severity. Besides, it is noted that driving in areas with traffic controls with low visibility will provide more safety (see Section 5.1), compared with normal visibility (>200 m). 

We also conclude that driving on road segments, rather than at intersections, would cause severe injuries in the case of an accident. According to Uddin and Huynh [7], crashes occurring on road segments are more serious than those at intersections, which conforms with the conclusion of this study. It is noted that driving on road segments with low visibility will also provide more safety (see Section 5.1), compared with normal visibility (>200 m). The driving environment also determines the injury severity. If the line of sight of the riders is only 50 m, according to the findings of this study, bicyclists generally do not sustain injuries. Night driving increases the probability of electric bicyclists’ severe injury, irrespective of the presence of streetlights. However, the presence of heterogeneity leads to underestimation, which means that night driving may lead to severe injuries, and streetlights cannot entirely compensate for the lack of sunlight at night. Thus, for better safety, this study suggests that headlights of electric bicycles should be required to be on during afternoon peak periods to provide more road illumination. Additionally, downtown riding increases the risk of severe injuries to electric bicycle riders (Table 4 and Table 5).

## 6. Discussion

Firstly, consider the heterogeneity observations of poor visibility as a factor influencing the injury severity. Driving on road segments instead of at intersections amid low visibility motivates electric bicyclists to drive cautiously, and thus prevent accidents that lead to severe injuries. This is a result of the segment having a relatively simple traffic organization and few accident-prone zones. Additionally, drivers tend to decelerate for effective braking during dangerous circumstances [13,51,52]. However, this opposes the findings of previous studies, which showed that segment driving was riskier than intersection driving [7,53]. This contradiction may indicate that poor visibility during the peak period influences the sensitivity of drivers to different road types with different levels of complexity, which warrants further research. On the other hand, driving in the traffic control area under poor visibility will reflect drivers’ diverse performances and then lead to different injury severities. The results suggest that traffic control approaches and facilities must consider this diversity. Consequently, separate approaches and facilities are required to handle traffic during peak periods under low visibility, including specialized signals that inform drivers of oncoming vehicles under haze or fog. Moreover, the results suggest that the 100–200 m visibility range decreases the probability of severe injury to electric bicyclists, but the reduction is markedly weakened when driving in areas with no nightlights. This indicates that 100–200 m visibility decreases the possibility of severe injury to electric bicyclists, albeit at a significantly lower rate in areas with no nightlights. This also means that a lack of sunlight due to haze during the afternoon peak periods reduces drivability and increases the risk of grave accidents. The presence of streetlights during the evening peak hours adversely affects the electric bicyclist’s driving performance and leads to severe injury rates fluctuating more. Therefore, we recommend proper road illumination during the evening peak traffic periods, especially under poor visibility. However, the varying effects of low visibility on injury severity during peak hours cannot be dismissed. The effect of low visibility on injury severity necessitates further investigation because it exhibits more uncertainty and variation than normal visibility [51,54,55,56].

Among the various types of automobiles, accidents involving passenger cars and trucks contribute to the increasing number of incidents causing severe injuries. Thus, we recommend restricting the movement of trucks and passenger cars during peak traffic periods, especially in a mixed traffic flow. Then, vehicular movement before a crash influences the injury severity of electric bicyclists. This suggests that during peak periods, different movement lanes should be separated and controlled strictly, especially for right-turn lanes, U-turn lanes, and bicycle lanes. Moreover, it is noted that driving on road segments with low visibility will also provide more safety. Thus, in clear weather, road segments should be better managed and regulated during peak traffic periods. Finally, night driving may lead to severe injuries, and streetlights cannot entirely compensate for the lack of sunlight at night. Thus, for better safety, this study suggests that headlights of electric bicycles should be required to be on during afternoon peak periods to provide more road illumination. A further novel finding is that although belonging into the green plant protection, the protective effects of green belt and trees are significantly different. In line with previous study [57], the most effective roadside protection lied on the Roadside Protection Trees with the magnificent marginal effect values. Nevertheless, the green belt has no significant impact on accident injuries during peak periods. Additionally, downtown riding will increase the risk of severe injuries to electric bicycle riders, which is intuitive. The reason for this may be the more complicated traffic scene in downtown areas [20]. With heterogeneity, this impact is emphasized, and new downtown traffic optimization concepts aimed at the peak hours should be proposed.

There are several limitations that should be considered when interpreting the results of this study. First, this study does not differentiate between morning and evening peak periods due to data limitation. It is possible that the two peak periods show different crash patterns, which deserve more investigation. We suggest that the police enforcement agency should include time period information when collecting crash data. Second, although we examined many sources of observed heterogeneity, some exogenous factors were ignored, such as the use of a reserved bus lane. Future work should check the impacts of those variables when related data is available. Third, several road features specific to electric bicycles were excluded, such as the presence of separate bike lanes. Special road features/signs warning electric bicycle riders may produce various effects and deserve more research, such as instrumented guardrails. Fourth, lighting and traffic accidents maintain a complicated interrelationship and this should be further addressed in future work. Fourth, different lighting conditions may reflect different types of traffic accidents, and more detailed types of lightning conditions should be further addressed on the traffic safety assessment in future work. Finally, in addition to traditional econometrics modeling, emerging approaches of machine learning such as fuzzy logic can be considered to address the heterogeneity of indicators and design new possibilities. Future research can compare the insights from the two study approaches, which help us better understand the crash mechanisms between electric bikes and cars.

## 7. Conclusions

The peak traffic period possesses unique traffic characteristics but is often dismissed in traffic safety research due to the heterogeneity of parameters. Therefore, traffic safety-related factors and methods to optimize relevant facilities during the peak traffic hours must be determined. People in China have adopted electric bicycles for commuting during peak traffic hours owing to their maneuverability; however, traffic data indicates high crash incident rates among electric bicycles. This study identified the factors influencing injury severity in electric bicyclists during rush hours. The characteristics of automobile drivers and bicyclists, vehicle maneuvers before the crash, and circumstances surrounding the accident were considered. Three mixed logit models considering the variations in parameter means and variances were constructed to provide insights into the potential sources of the heterogeneity.

The findings can be summarized as follows:

1. A vehicle taking a U-turn ahead of an electric bicycle is less likely to cause severe injuries to the rider. The vehicle turning right further decreases the possibility of electric bicyclists sustaining severe injuries than left-turning because the latter moves in the left lane.
2. The heterogeneity observations of poor visibility as a factor influencing injury severity disagree with those of previous studies. High visibility is not an absolute guarantee of less injury. Instead, it may present a potential risk of serious injury during peak periods. Therefore, to improve safety and lower the possibility of severe injuries, road segment control strategies must be modified to address the influence of high visibility during peak traffic hours.
3. Amid poor visibility, driving at night without streetlights and driving in areas of traffic control pose a greater risk of electric bicyclist injury.
4. There are significant differences between the protective effects of green belts and trees on two-wheelers during peak hours: the former have no significant impact on accident injuries while the latter is found to be the most effective roadside protection.

We recommend creating clear partitions between lanes and controlling them efficiently, especially right-turning and U-turn and bicycle lanes. Moreover, traffic control measures during peak traffic periods under low visibility must be reconsidered. For example, authorities could install specialized signals that inform the riders of oncoming vehicles amid haze. Importantly, roadside protection trees could be considered as the crucial roadside protective measure to reduce the risk of crashes. Specifically, apart from streetlights, headlights of electric bicycles must be required to be on during the evening peak traffic hours to prevent glare and excess brightness.

## Figures and Tables

**Table 1:** Summary of studies considering accidents in peak traffic periods.

Study	Methodology	Object of Study	Heterogeneity	Key Finding
[17]	Review	/	/	The study proposed to cope with the “excess” peak-hour demand for road space by constructing sufficient public transit facilities and shifting all the “excess” peak-hour demand there.
[18]	Multinomial logit model	Injury severity	/	In urban areas, crashes happened between 5 a.m. and 8 a.m. Application of the model can reduce the possibility of drivers suffering severe or fatal injuries.
[21]	Mixed logit model	Injury severity	/	In a single-vehicle model, accidents on rural highways during the busy harvest period may cause non-incapacitating injuries.
[3]	Mixed logit model	Injury severity	/	Different periods have different contributing factors to each degree of injury severity.
[7]	Mixed panel multinomial logit model	Traveler choices	√	Socioeconomic factors, work attributes, and trip characteristics (degree of flexibility) affect the traveler’s response during the peak traffic period.
[19]	Structural equation model	Vehicle movement	/	Weekday travel influences peak-hour travel more than weekend, and the choice of road and car types have different effects on peak-hour travels.
[6]	Mixed logit model	Injury severity	√	Different periods have different impacts on different degrees of injury severity.
[20]	Negative binomial regression and zero-inflated negative binomial regression	Crash frequency	/	Pedestrians are more likely to be hit by a vehicle if they cross signalized traffic light intersections during peak traffic hours. During the peak period, road segments with more bus stops are more likely to cause collisions between vehicles and pedestrians.
[5]	Mixed logit model	Injury severity	√	Crashes occurring during the morning peak hours were found to increase the probability of major injuries in sunny weather, whereas crashes occurring during the evening peak hours were found to increase the probability of major injury in snowy weather.

/ indicates the study does not consider the indicator’s heterogeneity; √ indicates the study considers the indicator’s heterogeneity.

**Table 2:** Summary of the literature about the traffic safety of electric bicycles (bicyclists).

Study	Methodology	Unique Factors	Heterogeneity	Key Findings
[22]	Accident reconstruction simulation	Head impact speed, time of head impact, and impact angle of bicyclists with vehicle impact speed, wrap-around distance, and throw-out distance	/	Wrap-around distance, head impact speed, time of head impact, head impact angle, and throw-out distance of bicyclists have a strong relationship with the vehicle impact speed. A higher vehicle impact speed puts the electric bicyclist at a higher risk of injury.
[23]	Historic prospective study	Population group, hospital resource utilization, discharge disposition, and injured body region	/	Arab children (aged 0–15) and young adults (aged 16–29) are at higher risk of e-bike accidents. E-bikers are at a greater risk of head and lower-extremity injuries. Consequently, they will require surgery, longer hospital stays, and visits to the rehabilitation center.
[24]	Simple chi-square statistics analysis and logit regression model	Gender, distance cycled/week, bicycle type, participants’ reported cause of accidents	/	Females are more prone to accidents on electric bikes than conventional ones, whereas males are equally prone to accidents on both bikes.
[10]	Retrospective study	Ethnicity, motorized device, nonmotorized device and type of impact	/	Electric bikes always cause mild injuries, which are mainly superficial wounds and upper- and lower-limb injuries.
[26]	Retrospective cohort study	Region, oral, and maxillofacial injuries, and hospital resource utilization	/	Electric bikers suffer mainly oral and maxillofacial injuries and pedestrians involved in electric bike crashes, who are mostly children and older people, suffer oral and maxillofacial injuries.
[25]	Multiple-factor conditional logistic regression	Marital status, electric bike type, and electric bikers’ behavior	/	Multiple-factor conditional logistic regression analysis of e-bike-related traffic crashes identified running red lights, drinking and riding, carrying adults while riding, turning without signaling, riding in the motor vehicle lane, prior crash history, and type of e-bike as possible risk factors for e-bike traffic crashes.
[14]	Main factor analysis	Collision objects, speed, driving direction, sight obstacle, and riders’ violation	/	Two-wheel electric vehicles are most prone to accidents when turning left. The most common collision object for two-wheel electric-vehicle riders are automobiles.
[27]	In-depth accident reconstruction and validated finite element model	Stress–strain performance, material of helmet outer shell, landing condition, and velocity of three parts of the human body before head impact	/	Electric bicyclist helmets not offering adequate protection increase the risk of injury.
[9]	Finite element model	Geometric and mass parameters of bicycle and electric two-wheeler and moving velocities of all parties and their initial relative position	/	The risk of head injury to electric bicyclists increases with the oncoming vehicle velocity. Riders with a larger stature have a higher chance of escaping head impact on the vehicle. In collision with a sedan or an SUV will cause electric bicyclists’ lower head injuries.

/ indicates that the study does not consider the indicator’s heterogeneity; SUV— sport utility vehicled.

**Table 3:** Descriptive statistics of key indicator variables (1 if variable is true; 0 otherwise).

Variable	Mean	Standard Deviation (SD)	Variable	Mean	SD
**Driver and Bicyclist Characteristics**			**Roadway and Environmental Characteristics**		
Male Vehicle Driver	0.91	0.29	Time of accident is a weekday	0.73	0.44
Male Electric Bicyclist	0.71	0.45	Roadway location is under traffic control	0.18	0.39
Electric Bicyclist Age Group < 18 years	0.36	0.48	Roadside protection is not provided	0.60	0.49
Electric Bicyclist Age Group 18–30 years	0.33	0.47	Roadside protections are trees	0.14	0.35
Electric Bicyclist Age Group 31–40 years	0.24	0.43	Roadside protections are green belts	0.13	0.34
Electric Bicyclist Age Group 41–50 years	0.07	0.25	Roadside protections are fences	0.07	0.26
Electric Bicyclist Age Group > 50 years	0.01	0.11	Roadside protections are truck escape ramps	0.05	0.22
Vehicle Driver Age Group 18–30 years	0.18	0.39	Roadside protections are protective piers	0.11	0.19
Vehicle Driver Age Group 31–40 years	0.20	0.40	Roadside protections are buffers	0.38	0.14
Vehicle Driver Age Group 41–50 years	0.24	0.42	Road surface condition is rough	0.99	0.11
Vehicle Driver Age Group > 50 years	0.36	0.48	Road surface is dry	0.89	0.31
Vehicle Driving Experience 1–5 years	0.22	0.41	Pavement structure is bituminous	0.92	0.27
Vehicle Driving Experience 6–10 years	0.26	0.44	Crash occurred in road segments	0.79	0.40
Vehicle Driving Experience 11–15 years	0.41	0.49	Road alignment is flat and straight	0.90	0.30
Vehicle Driving Experience > 15 years	0.11	0.32	Road type is general urban road	0.58	0.49
Intoxicated	0.16	0.64	Road type is graded highway	0.28	0.45
**Vehicle Characteristics**			Road type is urban expressway or another urban road	0.14	0.34
Vehicle Insured	0.99	0.10	Weather is sunny	0.78	0.41
Sedan	0.74	0.44	Weather is foggy	0.65	0.14
Passenger Car	0.06	0.24	Weather is cloudy	0.13	0.34
Truck	0.18	0.38	Weather is rainy	0.07	0.26
Motorcycle	0.02	0.15	Weather is snowy or covered with hail	0.01	0.11
* Abnormal	0.99	0.11	Visibility is more than 200 m	0.49	0.50
Overloaded	0.02	0.13	Visibility is 100–200 m	0.23	0.42
**Pre-crash Vehicle Movement Characteristics**			Visibility is 50–100 m	0.20	0.40
Go Straight	0.76	0.42	Visibility is less than 50 m	0.09	0.28
U-turn	0.02	0.16	Landform is plain	0.97	0.17
Turning Left	0.10	0.30	Lighting condition is daytime	0.71	0.46
Turning Right	0.11	0.32	Lighting condition is ‘streetlight at night’	0.20	0.40
No Braking	0.23	0.14	Lighting condition is ‘no streetlight at night’	0.07	0.26
Partial Braking	0.06	0.22	Lighting condition is natural light of dawn or dusk	0.02	0.15
Entire Braking	0.18	0.66	Location of accident is downtown	0.45	0.50
Throttle Loose	0.05	0.24	Construction area	0.09	0.21

Vehicles with poor braking/braking failure/steering issues/illuminance issues/other mechanical issues; * represents the baseline of the category.

**Table 4:** Different mixed logit models (with/without heterogeneity) results.

Variable	Mixed Logit		
	No Mean–Variance Heterogeneity	Mean Heterogeneity	Mean–Variance Heterogeneity
	Coefficient (*t*-Statistic)	Coefficient (*t*-Statistic)	Coefficient (*t*-Statistic)
Constant [I]	5.428 ***(8.57)	5.428 *** (8.57)	5.473 *** (9.12)
Constant [I+]	−3.957 *** (−8.62)	−3.957 *** (−7.95)	−3.716 *** (−7.57)
Constant [I++]	−2.854 *** (−18.47)	−2.854 *** (−18.47)	−2.811 *** (−19.04)
**Driver and Bicyclist Characteristics**			
Female Electric Bicyclist [I]	−1.228 *** (3.12)	−1.228 *** (3.12)	−1.237 *** (3.04)
**Vehicle Characteristics**			
Passenger Car [I+]	0.653 ** (2.46)	0.653 ** (2.46)	0.701 ** (2.56)
Passenger Car [I++]	1.122 *** (6.08)	1.125 *** (6.05)	1.408 *** (4.79)
Truck [I+]	1.125 *** (6.58)	1.196 *** (6.46)	1.187 *** (6.34)
Truck [I++]	1.756 *** (11.78)	1.833 *** (11.56)	1.825 *** (10.89)
Motorcycle [I]	−1.288 *** (−3.56)	−1.455 *** (−4.05)	−1.455 *** (−3.88)
**Pre-crash Vehicle Movement Characteristics**			
U-turn [I+]	−1.857 *** (−3.07)	−1.946 *** (−4.05)	−2.105 *** (−3.94)
U-turn [I++]	−1.887 ** (−2.22)	−1.889 ** (−2.44)	−1.890 ** (−2.42)
Turning Left [I+]	−2.055 *** (−5.02)	−2.277 *** (−4.89)	−2.028 *** (−4.88)
Turning Left [I++]	−1.588 *** (−4.02)	−1.276 *** (−3.48)	−1.426 *** (−4.02)
Turning Right [I+]	−1.725 *** (−5.20)	−1.701 *** (−5.16)	−1.770 *** (−6.42)
Turning Right [I++]	−0.653 ** (−2.12)	−0.653 ** (−2.12)	−0.652 ** (−2.08)
**Roadway and Environmental Characteristics**			
Traffic Control [I+]	−0.725 *** (−3.22)	−0.728 *** (−3.37)	−0.806 *** (−3.91)
Roadside Protection Trees [I+]	−0.988 *** (−4.29)	−0.993 *** (−3.43)	−1.021 *** (−3.84)
Roadside Protection Fences [I+]	−1.428 *** (−4.01)	−1.458 *** (−4.15)	−1.559 *** (−4.15)
Road Segments [I+]	2.048 *** (4.26)	2.125 *** (4.12)	2.218 *** (4.86)
Flat and Straight Road Alignment [I+]	−1.701 *** (3.04)	−1.628 *** (3.22)	−1.112 *** (4.07)
Graded Highway [I+]	0.480 ** (2.41)	0.491 ** (2.41)	0.485 ** (1.95)
Graded Highway [I++]	0.855 *** (4.94)	0.877 *** (4.85)	0.827 *** (4.88)
Urban Expressway or another Urban Road [I+]	0.852 *** (3.97)	0.565 *** (3.48)	0.786 *** (4.05)
Visibility < 50 m [I]	−0.528 ** (−2.42)	−0.701 ** (−2.39)	−0.897 ** (−2.37)
Streetlights at Night [I+]	0.398 ** (2.17)	0.527 ** (2.11)	0.242 ** (2.19)
No Lights at Night [I+]	0.958 ** (2.13)	0.727 ** (2.34)	0.672 ** (2.48)
Downtown Driving [I+]	1.424 *** (6.12)	1.486 *** (5.78)	1.271 *** (6.01)
**Random Parameters (Normal Distribution)**			
Visibility 50–100 m [I+]	−2.181 ** (−2.25)	−2.117 ** (−2.14)	−2.331 ** (−2.21)
*SD for random parameter*	2.348 ** (2.32)	2.294 ** (2.14)	2.581 ** (2.49)
Visibility 100–200 m [I+]	−1.797 ** (−2.36)	−3.275 ** (−2.32)	−3.127 ** (−2.45)
*SD for random parameter*	2.023 ** (2.24)	3.946 ** (2.70)	4.037 ** (3.15)
**Heterogeneity in Means of the Random Parameters**			
Visibility 100–200 m: Traffic Control [I+]		1.626 ** (1.95)	1.418 ** (2.13)
Visibility 100–200 m: No Lights at Night [I+]			3.067 ** (2.05)
Visibility 50–100 m: Road Segments [I+]		−1.347 ** (−2.21)	
**Heterogeneity in Variances of the Random Parameters**			
Visibility 100–200 m: Traffic Control [I+]			0.568 ** (2.01)
Visibility 100–200 m: No Lights at Night [I+]			0.732 * (1.20)

(Parameter defined for: [I] minor injury, [I+] severe injury and [I++] fatal injury). No injury is the severity outcome baseline; its severity function is constrained to zero. Injury includes minor injury, severe injury, and fatality. Each variable is set as a dichotomous variable. 1 indicates the variable is true, and 0 otherwise. Significance codes: * *p* < 0.1, ** *p* < 0.05, *** *p* < 0.01.

**Table 5:** Marginal effects for different mixed logit models (with/without heterogeneity consideration).

Variable	Mixed Logit		
	No Mean–Variance Heterogeneity	Mean Heterogeneity	Mean–Variance Heterogeneity
**Driver and Bicyclist Characteristics**			
Female Electric Bicyclist [I]	−0.0137	−0.0134	−0.0255
**Vehicle Characteristics**			
Passenger Car [I+]	0.2711	0.2715	0.1533
Passenger Car [I++]	0.0238	0.0233	0.0114
Truck [I+]	0.0347	0.0355	0.0589
Truck [I++]	0.0433	0.0412	0.0407
Motorcycle [I]	−0.0291	−0.0344	−0.0308
**Pre-crash Vehicle Movement Characteristics**			
U-turn [I+]	−0.0472	−0.0564	−0.0566
U-turn [I++]	−0.0085	−0.0085	−0.0074
Turning Left [I+]	−0.0587	−0.0592	−0.0688
Turning Left [I++]	−0.0472	−0.0470	−0.0481
Turning Right [I+]	−0.0905	−0.1028	−0.1033
Turning Right [I++]	−0.0522	−0.0623	−0.0688
**Roadway and Environmental Characteristics**			
Traffic Control [I+]	−0.0804	−0.0910	−0.0912
Roadside Protection Trees [I+]	−0.1228	−0.1220	−0.1181
Roadside Protection Fences [I+]	−0.0523	−0.0412	−0.0404
Road Segments [I+]	0.0805	0.0927	0.0933
Flat and Straight Road Alignment [I+]	−0.0711	−0.0659	−0.0783
Classified Highway [I+]	0.0023	0.0021	0.0133
Classified Highway [I++]	0.0112	0.0110	0.0129
Urban Expressway or another Urban Road [I+]	0.0291	0.0284	0.0199
Visibility < 50 m [I]	−0.0672	−0.0665	−0.0638
Streetlights at Night [I+]	0.1862	0.1877	0.1928
No Lights at Night [I+]	0.3486	0.3522	0.3697
Downtown Driving [I+]	0.0632	0.0703	0.0710
**Random Parameters (Normal Distribution)**			
Visibility 50–100 m [I+]	−0.1824	−0.1810	−0.1776
Visibility 100–200 m [I+]	−0.1791	−0.1774	−0.1739

Parameter defined for: [I] minor injury, [I+] severe injury and [I++] fatal injury). Each variable is set as a dichotomous variable. 1 represents the variable is true and 0 represents otherwise. All the marginal effects in the no injury function are implicit and have not been reported due to space constraints. Marginal effects are presented as decimals.

**Table 6:** Model comparison.

Indicators	No Mean–Variance Heterogeneity	Mean Heterogeneity	Mean–Variance Heterogeneity
Number of Observations	2141	2141	2141
Log Likelihood with Constants Only	−1947.61	−1947.61	−1947.61
Log Likelihood at Convergence	−1625.30	−1611.70	−1602.48
Adjusted McFadden—ρ^2^	0.564	0.640	0.642
Akaike Information Criterion	3300.7	3299.3	3153.8
Bayesian Information Criterion	3288.5	3397.3	3530.7

## Data Availability

The dataset used in this research are available upon request from the corresponding author. The data are not publicly available due to restrictions i.e., privacy or ethical.
